# Complete plastome sequence of *Prunus hypoxantha* (Rosaceae), a species endemic in west of China

**DOI:** 10.1080/23802359.2020.1861995

**Published:** 2021-01-20

**Authors:** Jun-Ru Wang, Yu-Hui Guo, Jin-Qiu Yang, Ru-Chang Tong, Zhen Cao, Liang Zhao

**Affiliations:** a College of Life Sciences, Northwest A&F University, Yangling, China; b College of Innovation and Experiment, Northwest A&F University, Yangling, China

**Keywords:** *Prunus hypoxantha*, plastome, genome structure, Rosaceae

## Abstract

The Chinese endemic species *Prunus hypoxantha* is of great importance biogeographically, and is distributed in the edge of the Qinghai-Tibetan Plateau. Here, the complete chloroplast genome sequence of *P. hypoxantha* was assembled based on the Illumina reads. The complete plastome is 158,740 bp in length, with a large single-copy (87,206 bp) region, a small single-copy (18,884 bp) region, and two inverted repeat (26,325 bp) regions. The plastome contains 134 genes. Complete chloroplast genome sequence of *P. hypoxantha* will provide irreplaceable information in rebuilding the evolutionary history of the *Maddenia* clade.

*Prunus hypoxantha* (Koehne) J. Wen is a member of the *Maddenia* clade of *Prunus*. It grows in alpine forests of western to central China (Kalkman 2004; Wen and Shi 2012). As its distribution area includes the east most edge of the Qinghai-Tibetan Plateau (QTP), its phylogenetic research may be helpful to explain the speciation on QTP. Here, the complete plastid genome of *P. hypoxantha* (GenBank accession number: MK911761) was assembled by using the genome skimming approach (Zimmer and Wen 2015).

Fresh leaves of *P. hypoxantha* was sampled from Caiyuanzigou, Kangding Country, Sichuan Province, China (101°57’47′’E, 30°03’15′’N, alt. 2705 m), where the type specimen was collected, and dried with silica gel. A voucher specimen (ZL201708002) was deposited in the Herbarium of Northwest A&F University (WUK), China. Total genomic DNA was extracted with CTAB method (Doyle and Doyle 1987). A 500-bp DNA TruSeq Illumina (Illumina Inc., San Diego, CA, USA) sequencing library was constructed and then sequenced on Illumina Miseq platform (Illumina, San Diego, CA, USA). Reads of the chloroplast genome were assembled with GetOrganelle (Jin et al. 2020). The complete cp genome was annotated using PGA (https://github.com/quxiaojian/PGA) and corrected with DOGMA (Wyman et al. 2004). We submitted all the newly sequence data in raw format (fastq) and with the SRA accession (SRR12920654).

The complete plastome sequence of *P. hypoxantha* is 158,740 bp in length. The length of each inverted repeat (IR) is 26,325 bp, while the length of the large single-copy (LSC) and the short single-copy (SSC) is 87,206 bp and 18,884 bp, respectively. 134 genes were found in this plastome, 87 of them are unique protein-coding genes, while 39 are unique tRNA genes and 8 are unique rRNA genes. Most of the genes have a single copy, while nine tRNA genes (*trnI-CAU*, *trnL-CAA*, *trnV-GAC*, *trnI-GAU*, *trnA-UGC*, *trnR-ACG*, *trnR-CCG*, *trnN-GUU* and *trnG-GCC*), 5 protein-coding genes (*rpl2*, *rpl23*, *ycf2*, *ndhB* and *rps7*) and all the four rRNA genes are duplicated. The protein-coding gene *rpl2* has three copies. The overall G/C content in the plastome is 36.6%.

Totally 13 species (including 11 of Rosaceae, and *Broussonetia papyrifera* and *Morus cathayana* of Moraceae as outgroups) were used as outgroups to obtain the phylogenetic position of *P. hypoxantha* by RAxML. *P. hypoxantha* as well as the four other *Prunus* species forms a clade, which supports that *P. hypoxantha*, once belongs to a single genus *Maddenia*, should be a member of *Prunus* (Figure 1). Such result was consistent with several other molecular studies (Wen et al. 2008). The complete chloroplast sequence of *P. hypoxantha* will provide useful resource for further phylogenetic and biogeographic study.

## Data Availability

The genome sequence data that support the findings of this study are openly available in GenBank of NCBI at [https://www.ncbi.nlm.nih.gov](https://www.ncbi.nlm.nih.gov/) under the accession no. MK911761. The associated BioProject, SRA, and Bio-Sample numbers are PRJN 671803, S12920654, and S16552112 respectively. The ML phylogenetic tree based on 11 complete chloroplast genomes plus two species as outgroups.
